# Association of exercise-induced autophagy upregulation and apoptosis suppression with neuroprotection against pharmacologically induced Parkinson's disease

**DOI:** 10.20463/jenb.2018.0001

**Published:** 2018-03-31

**Authors:** Yong chul Jang, Dong Joo Hwang, Jung Hoon Koo, Hyun Seob Um, Nam Hee Lee, Dong Cheol Yeom, Youngil Lee, Joon Yong Cho

**Affiliations:** 1. Exercise Biochemistry Laboratory, Korea National Sport University, Seoul Republic of Korea; 2. Department of Exercise Prescription, Kon-Yang University, Nonsan Republic of Korea; 3. Department of physical education, Dan Kook University, Cheonan Republic of Korea; 4. Molecular and Cellular Exercise Physiology Laboratory, Department of Exercise Science and Community Health, University of West Florida, Pensacola Republic of Korea

**Keywords:** Parkinson’s disease; α-synuclein, autophagy, apoptosis, treadmill exercise

## Abstract

**[Purpose]:**

We investigated whether treadmill exercise (TE)-induced neuroprotection was associated with enhanced autophagy and reduced apoptosis in a mouse model of pharmacologically induced Parkinson's disease (PD).

**[Methods]:**

PD was induced via the administration of 1-methyl-4-phenyl-1,2,3,6-tetrahydropyridine (MPTP). C57BL/6 male mice were randomly assigned to the following three groups: control (C57BL, *n*=10), MPTP with probenecid (MPTP/C, *n*=10), and MPTP/ C plus exercise (MPTP-TE, *n*=10). The MPTP-TE mice performed TE training (10 m/min, 60 min/day, 5 days/week) for 8 weeks. The rotarod test was used to assess motor function.

**[Results]:**

TE restored MPTP/P-induced motor dysfunctionand increased tyrosine hydroxylase levels. Furthermore, TE diminished the levels of α-synuclein (α-syn), a neurotoxin; modulated the levels of autophagy-associated proteins, including microtubule-associated protein 1 light chain 3-II, p62, BECLIN1, BNIP3, and lysosomal-associated membrane protein-2, which enhanced autophagy; inhibited the activation of proapoptotic proteins (caspase-3 and BAX);and upregulated BCL-2, an antiapoptosis protein.

**[Conclusion]:**

Taken together, these results suggested that the TE-induced neuroprotection against MPTP-induced cell death was associated with enhanced autophagy and neuronal regeneration based on the findings of inhibited proapoptotic events in the brains of the TE-trained animals.

## INTRODUCTION

Parkinson’s disease (PD) is a progressive neurodegenerative disorder characterized by motor dysfunction, including bradykinesia, tremor, rigidity, and postural instability^2^ The main cause of PD is the progressive loss of dopaminergic neurons in the substantia nigra (SN) pars compacta (SNpc) and the accumulation of Lewy bodies, which are primarily composed of alpha-synuclein (α-syn) proteins. Although normal α-syn functions are necessary for neurotransmission, dopamine metabolism, and synaptic vesicle release^3^ the aberrant aggregation of α-syn contribute to mitochondrial dysfunction and cellular oxidative stress, which then triggers neuronal death in PD^4-5^.

Although the exact mechanisms of PD remain unclear, recent studies have suggested that autophagy plays a crucial role in the pathogenesis and progression of the neurodegenerative disease^6^. Autophagy is a lysosome-dependent intracellular catabolic process that removes damaged/misfolded proteins and small cellular organelles, such as mitochondria. Thus, autophagy has emerged as one of the most critical mechanisms in cell survival regulation^7^. More importantly, because autophagy plays a key role in the elimination of abnormal α-syn proteins^8^, autophagy interference contributes to the neuronal cell death resulting from α-syn-induced toxicity in dopaminergic neurons in PD^7^. Given that basal levels of autophagy must be maintained for proper brain function and that impaired autophagy contributes to the progression of PD, positive modulations of autophagy mightserve as potential therapeutic strategiesfor PD.

Autophagy is assessed by measuring the levels of autophagy markers, such asmicrotubule-associated protein 1 light chain 3 (LC3) and p62 proteins. Once autophagy is activated, LC3-1 becomes lipidated with phosphatidylethanolamine, which results in the production of LC3-II^9^. Therefore, the conversion of LC3-I to LC3-II is thought to reflect increased autophagy. However, LC3-II also accumulates in response to impairments in the fusion of autophagosomes and lysosomes. Therefore, to ensure that any observed changes in LC3- II reflect autophagy processes, the levels of p62 protein, an autophagy adaptor,are concomitantly measured ^10^. Because p62 relays polyubiquitinated proteins to autophagosomes for lysosome-mediated degradation, decreased levels of p62 are thought to reflect enhanced autophagy^11^.

Regular treadmill exercise (TE) confers protection against neurodegenerative diseases, including PD and Alzheimer’s disease, by enhancing neurotrophic factors that promote neurogenesis, reduce inflammation, and improve mitochondrial function^12^. The positive outcomes of physical exercise correlate highly with improved motor performance, muscle strength, and balance in patients with PD. In addition, recent PDanimal model studies have shown that TEinduces neuroprotection through a protective mitochondria phenotype and the increased expression of tyrosine hydroxylase (TH), a key enzyme in dopamine biosynthesis^13-14^.

However, the molecular mechanisms underlying the neuroprotective effects of exercise on dopaminergic neurons remain unknown. Because autophagy plays a critical role in the removal of aberrant α-syn, which is known to cause neurotoxicity, and exercise is a potent inducer of autophagy in tissues, such as skeletal muscles and hearts^15-16^, we hypothesized that TE would increase autophagy in the SNpc of mice with pharmacologically induced PD, which would then be associated with a decrease in α-syn and cell death.

## METHODS

### Animals

**Fig.1.**
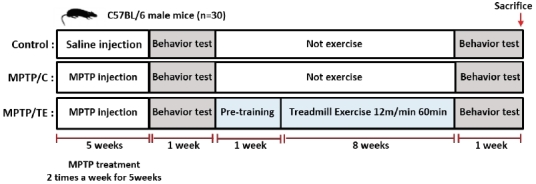
Overall experimental design

Male C57BL/6J mice (7 weeks old) were purchased from Samtako Bio Korea Co., Ltd. (Gyeonggi, Korea) and housed in a controlled environment with a 12:12-h darklight cycle at 22 ± 2°C and 50% relative humidity. Food and water were provided ad libitum. The animals were randomly divided into the following three groups: control-treated saline mice (C57BL, *n*=10), 1-methyl-4-phenyl-1,2,3,6-tetrahydropyridine (MPTP) with probenecid-treated mice (MPTP/C, *n*=10), and MPTP/P-treated TE mice (MPTP-TE, *n*=10) (Fig. 1). All procedures were approved by the Institutional Animal Care and Use Committees of Korea National Sport University (KNSU-IACUC-2014-02).

### Chronic mice model of PD

To induce chronic PD, MPTP/P-treated mice (8 weeks old, *n*=20) were administeredintraperitoneal injections of a total of 10 doses of MPTP (25 mg/kg, Sigma-Aldrich Corporation, St. Louis, MO, USA) and probenecid (250 mg/kg, Sigma-Aldrich Corporation) for five weeks at 3.5-day intervals,while control mice were administered saline^17^.

### TE

After the five-week MPTP treatment, the MPTP-treated exercise mice (3months old, *n*=10) were familiarized with TE (8m/min, 30 min/day) for five days, after which the animals performed regular TE training (10 m/min, 60 min/day, 5 days/week) for eight weeks.

### Rotarod test

We performed rotarod tests (JD-A-07RA5, Jeung Do Bio & Plant Co., Ltd., Seoul, Korea) to evaluate motor function after the MPTP treatment and eight weeks of TE. A mouse was placed on a rotating rod, and the speed was gradually increased to 40 rpm. Each mouse performed two trials, and the latency time until a fall was recorded. The maximum trial time was limited to 300 s/trial.

### Tissue preparation

After the rotarod tests, all mice were anesthetized with 50mg/kg of Zoletil®50 (Virbac, Carros, France). Six mice/group were randomly assigned for use in the western blot analyses. Each brain was rapidly excised, the SN was isolated, and the tissue was then stored at -80°C until the analyses. For the immunofluorescent microscopy, four mice/group were perfused with 50 mM phosphate-buffered saline (PBS) and then postfixed for 30 min with 4% paraformaldehyde in 0.1 M sodium phosphate buffer (pH 7.4).

### Western blotting

The brain tissue (SN) was homogenized in radioimmunoprecipitation assay lysis buffer, and the protein concentrations were determined with a Bradford assay^18^. The proteins (30μg) were separated by 10–12% sodium dodecyl sulfate-polyacrylamide gel electrophoresis, transferred to a polyvinylidene fluoride membrane, and blocked with 5% skim milk for 1 h at ambient temperature. The membranes were incubated overnight at 4°C with the following primary antibodies (all dilutions, 1:1,000): α-syn (BD Biosciences, San Jose, CA, USA); TH (MilliporeSigma, Burlington, MA, USA); LC3 A/B (Abcam plc, Cambridge, UK); p62, BECLIN1, BNP3, and Caspase-3 (Cell Signaling Technology, Inc., Danvers, MA, USA); and BAX, BCL2, and α-tubulin (Santa Cruz Biotechnology, Inc., Dallas, TX, USA). After overnight incubations with each primary antibody, the membranes were washed with PBS-Tween 20 and incubated with the proper secondary antibodies (horseradish peroxidase-conjugated goat anti-rabbit, rabbit anti-goat, or goat anti-mouse;all dilutions, 1:5,000). The proteins of interest were identified using the ECL western blotting detection system (Santa Cruz Biotechnology, Inc.), and the density of each protein band was assessed using ChemiDoc XRS software (Bio-Rad Laboratories, Inc., Hercules, CA, USA). To quantify protein levels, the levels of expression of the proteins in the CON group were normalized to 1,and the protein levels in the other groups werethen calculated as a percentage of the levels of the CON group.

### Immunofluorescent microscopy

The mice brains were sectioned at a thickness of 30μm using a sliding cryostat (Leica Biosystems GmbH, Wetzlar, Germany). The sections were rinsed three times in 0.01 M PBS for 10 min, permeabilized with 0.2% Triton X-100, and blocked with 10% normal donkey serum for 1 h at ambient temperature. After the blocking, the sections were incubated with anti-TH (1:200, MilliporeSigma) overnight at 4°C. The tissue sections were washed in 0.01 M PBS and incubated with the Alexa 488-conjugated donkey anti-rabbit secondary antibody (Jackson ImmunoResearch Laboratories, Inc., West Grove, PA, USA) for 2 h at ambient temperature. After washingin PBS, the sections were mounted on slides,covered with coverglass and Vectashield (Vector Laboratories, Inc., Burlingame, CA, USA), and examined using an immunofluorescent microscope (Leica Microsystems GmbH, Wetzlar, Germany). The number of TH-positive cells was measured by manually counting each photographed image at a 100× magnification as described previously^19^.

### Statistical analysis

All statistical analyses were performed with SPSS (version 18.0;IBM Corporation, Armonk, NY, USA). The data were analyzed usingindependent t-tests or one-way analysis of variance (ANOVA), which was followed by Bonferroni posthoc teststo determine the group differences. The data are presented as mean ± standard error of the mean. P values less than 0.05 were considered statistically significant.

## RESULTS

### TE improved motor function in mice with pharmacologically induced PD

**Fig.2.**
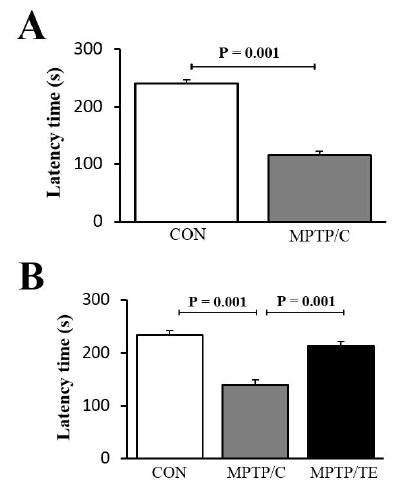
The effects of treadmill exercise on motor balance and coordination. A. Latency time after the administration of 1-methyl-4-phenyl-1,2,3,6-tetrahydropyridine (MPTP). B. Latency time after eight weeks of treadmill exercise (n=10/group). The values are presented as mean ± standard error of the mean (SEM).

Because the loss of dopaminergic neurons results in degenerative impairmentsin motor function, we evaluated the animals’ motor functions using the rotarod test. First, five weeksafter the MPTP treatment, the MPTP/C group exhibited significantly decreased retention times compared with those of theCON group (independent t-test, t=15.60, df=16.47, p=0.001, Fig. 2A). Next, we investigated whether TE improved the motor functions impaired by MPTP treatment. As expected, the eight weeks of exercise intervention significantly increased the latency time in the MPTP-TE group [F(2,27)=29.73, *p*= 0.001, one-way ANOVA, Fig. 2B].

### TE suppressedα-syn accumulation and activated autophagy proteins in mice with pharmacologically induced PD

**Fig.3.**
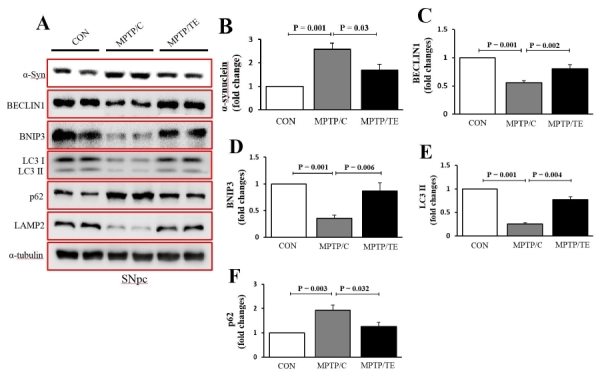
The effects of treadmill exercise on the expression of alpha-synuclein (α-syn) and autophagy proteins in the substantia nigra. A. Representative western blot data (n=6/group). B-F. Quantification of the levels of LC3-II, p62, BECLIN1, BNIP3, and LAMP2. α-tubulin served as a marker of equal loading. The values are presented as mean ± SEM.

Aberrant α-syn accumulation is linked to cell death and, thus, the loss of TH. Our data showed that MPTP treatment increased α-syn levels whileControl+Exercise remarkably diminished the α-syn levels in the MPTP-mice [F(2,17)=13.46, *p*= 0.001, one-way ANOVA, Fig. 3A, B]. To evaluate the effects of TE on autophagy flux levels, we analyzed the levels of the autophagy inducers, BECLIN1 and BNIP3. Control+Exercise upregulated BECLIN1 and BNIP3 in the MPTP/P-mice [BECLIN1: F(2,17)=27.08, p= 0.001; BNIP3: F(2,17)=12.27, *p*= 0.001, one-way ANOVA, Fig. 3A, C, and D]. In addition, we analyzed the levels of LC3 II and p62. Control+Exerciseincreased LC3 II levels and decreased p62 levels in the MPTP-mice [LC3 II: F(2,17)=80.40, *p*= 0.001; p62: F(2,17)=8.61, *p*= 0.01, one-way ANOVA, Fig. 3A, E, and F]. We next examined the expression of lysosomal-associated membrane protein-2 (LAMP2), which is a lysosomal biosynthesis marker. Our data showed that the LAMP2 levels in the MPTP/P-mice were remarkably decreased, whileControl+Exercise prevented the MPTP/P-induced LAMP2 degradation [F(2,17)=13.68, *p*= 0.001, one-way ANOVA, Fig. 3A, G].

### TE increased TH expression in theSNofmice with pharmacologically induced PD

**Fig.4.**
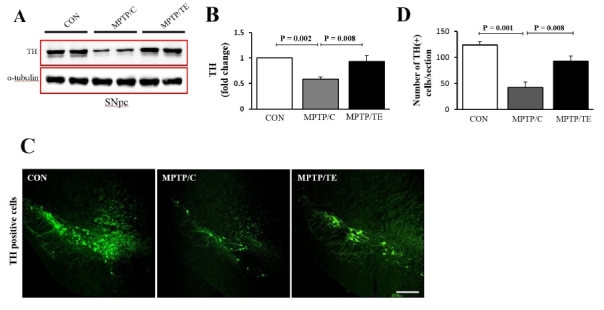
The effects of treadmill exercise on tyrosine hydroxylase (TH) expression in the substantia nigra. A. Representative western blot data (n=6/group). B. Quantification ofTH levels. α-tubulin served as a marker of equal loading. C-D. Representative immunofluorescent image of TH in the substantia nigra (n=4/group). Scale bars: 200μm. The values are presented as mean ± SEM.

TH is a key enzyme in dopamine biosynthesis. Given that TH levels are associated with dopaminergic neuron number, we measured TH levels using western blotting. Our data showed that TH levels remained decreased in the MPTP/C-mice, whileControl+Exerciseincreased TH levels to control levels [F(2,17)=10.66, p= 0.001, one-way ANOVA, Fig. 4A, B]. In addition, we performed a photomicrograph analysis and confirmed that Control+Exercisesignificantly increased the number of TH-positive neurons in the MPTP/P-induced PD mice [F(2,11)=97.08, *p*= 0.001, one-way ANOVA, Fig. 4C, D].

### TE decreased apoptosisin mice with pharmacologically induced PD

**Fig.5.**
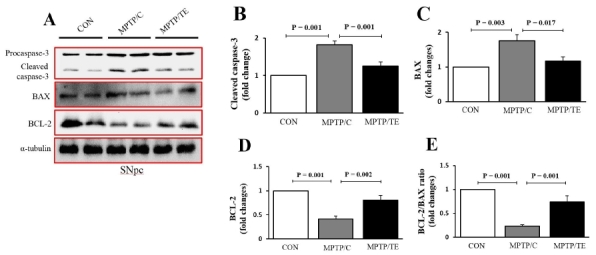
The effects of treadmill exercise on the expression of apoptosis-associated proteins in the substantia nigra. A. Representative western blot analysis (n=6/group). B-E. Quantification of caspase-3, BAX, BCL-2, and BCL-2/BAX ratio levels. α-tubulin served as a marker of equal loading. The values are presented as mean ± SEM.

Apoptosis is one of the main causes of the loss of dopaminergic neurons. To examine whether TE affected the expression of apoptosis-related proteins, we performed western blotting. The western blot data showed that MPTP treatment increased the levels of cleaved caspase-3, which is a cell death executioner, whileControl+Exercise significantly decreasedthe levels close to control levels [F(2,17)=20.92, *p*= 0.001, one-way ANOVA, Fig. 5A, B]. We examined two other effectors of apoptosis: Bax and BCL-2. The MPTP/P-mice exhibited significantly increased Bax levels and decreased Bcl-2 levels,and-Control+Exercise prevented the upregulation of Baxand increased Bcl-2, thus resulting in an increased Bcl-2/Bax ratio [Bax: F(2,17)=9.46, p= 0.01; Bcl-2: F(2,17)=20.53, *p*= 0.001; Bcl-2/Bax ratio: F(2,17)=28.50, *p*= 0.001, one-way ANOVA, Fig. 5A, C-E].

**Table 1.** Spearman correlations of therotarod test results and levels of markers in PD. Abbreviations: α-syn,α synuclein; TH, tyrosine hydroxylase (*p< 0.05)

	Rota-rod	α-syn	TH	LC3-II
Rota-rod	1.00			
α-syn	-0.811*	1.00		
TH 0.665*	-0.776*	1.00		
LC3-II	0.88*	-0.694*	0.823*	1.00

## DISCUSSION

In the present study, we examined the potential mechanisms underlying TE-induced neuroprotection against pharmacologically induced PD, and we obtained several interesting findings. First, TE improved motor function, which was accompanied by increased TH levels. Second, TE suppressed the MPTP/P-induced increase of α-syn, which is associated with increased autophagy. Third, TE mitigated apoptosis activation. Taken together, these results suggested thatTE-induced neuroprotection against PD was linked to exercise-induced autophagy promotion. Thus, the present study demonstrated that, as has been observed in other tissues, regular TE was a potent inducer of autophagy in the SNpc and that exercise-induced autophagy was associated with the suppression of α-syn accumulation and cell death.

MPTP is commonly used to produce animal models of PDbecause it closely mimics several PD symptoms and selectively activates toxic responses in the dopaminergic neurons^20^. MPTP inhibits the activity of the electron transport chain complex 1, which results in decreased ATP production, oxidative stress, and, ultimately, dopaminergic cell death. Additionally, probenecid delays the urinary excretion and neuronal clearance of MPTP, and, thus, its concurrent use with MPTP has been reported to be effective for inducing dopaminergic neuron degeneration in the SNpc^21^.

The rotarod test has been previously used to assess motor balance and coordination in the MPTP/P-induced mouse model^22^. In the present study, five weeks of MPTP/P administration decreased the latency time on the rod compared to that in the control group.However, TE rescued motor function from this MPTP/P-induced motor dysfunction. Consistently, other studies have also shown that exercise improves motor function in PD animal models and in patients with PD^23-24^. Many of the symptoms of the movement disorders in PD are caused by the death of dopaminergic neurons in the SNpc. TH, which is the rate-limiting enzyme in dopamine biosynthesis, is considered a marker of the integrity of dopaminergic neurons^25^. Furthermore, there is growing evidence that TE-induced neuroprotection in PD animal models correlates with the increased expression of TH proteins^17,26^. The present study also demonstrated that TE upregulated TH levels in the SNpc, thus confirming the current body of literature. These results clearly showed that the TE-induced restoration of motor function was linked to the mitigation of the dopaminergic neuronal loss caused by PD.

The abnormal accumulation of α-syn in dopaminergic neurons can directly trigger neuronal cell death in PD. Thus, recent studies have focused on how to reduce or eliminate the α-synaggregation as a potential therapeutic strategy that targets α-syn. In the present study, we observed that MPTP/P treatment increased α-syn accumulation in the SNpc; however, TE significantly reduced the levels of α-syn compared to that in the MPTP/C group. These results were consistent with our previous results of the suppression of α-syn expression by TE^13^. These resultssuggested that the neuroprotective effects of TE are regulated by α-syn modulation, which eventually prevents neuronal loss. However, the molecular mechanisms underlying how TE regulates α-syn levels in PD remain unknown. Nevertheless, recent studies have implicated autophagy as a potential mechanism of α-syn degradation that leads to neuroprotection ^27-9^.

The pivotal role of autophagy is to sustain cell survival and homeostasis via the clearance of damaged proteins and dysfunctional cellular organelles. Therefore, autophagy dysfunction plays a central role in the pathogenesis and progression of PD. Interestingly, a recent study has reported that accumulated α-syn contributes to dysfunctional autophagosome formation and impaired lysosomal function, both of which negatively affect the overall autophagy process^30^. Furthermore, Marques-Aleixo et al. ^31^have reported that TE in rats enhances autophagy signaling in the brain cortex and cerebellum. However, it is currently unknown whether TE regulates autophagy in a mouse model of PD, especially in the SNpc.

BECLIN1 and BNIP3 are proteins involved in autophagy. BECLIN1 is an essential marker of autophagy initiation, and BNIP3 acts as a potent inducer of autophagy by directly interacting with LC3^32^. We observed that the MPTP/P-treated mice had significantly decreased levels of BECLIN1 and BNIP3. In contrast, the TE group had increased levels of both proteins. We believe that the upregulation of these autophagy proteins, which reflects increased autophagy flux, is a key event in the proper autophagy process. The present results showed for the first time that TE increased the levels of LC3-II, a marker of autophagosome formation, and reduced the levels of p62 proteins, thus suggesting that TE promotes autophagy flux. Although we did not conduct a thorough mechanistic experiment ofTE-induced autophagy, our resultsstill suggest that TE-induced autophagy promotion prevents α-syn accumulation and the resulting α-syn-mediated autophagy inhibitory effects. However, because very little research has been conducted on this, further studies are needed to determine the exact mechanisms of exercise-mediated α-syn suppression.

Lysosomal fusion activities critically rely on LAMP2 proteins. A recent study has shown that LAMP2 is important for enabling lysosomes to degrade α-syn proteins^27^. Xilouri et al. ^33^have shown the importance of LAMP2 by demonstrating that the inhibition of LAMP2 results in α-syn accumulation as well as a progressive loss of dopaminergic neurons in the SNpc. In the present study, we observedthat TE upregulated LAMP2, while MPTP/P-treatment decreased its expression. These results suggested that TE enhanced the fusion process between autophagosomes and lysosomes, thus expediting α-syn clearance.

We further evaluated whether improved motor functions were associated with TE-induced modulations of α-syn, TH, and LC3-II. Our results suggested thatα-syn levelsnegatively correlated with the latency times on therotarod test, while TH and LC3-II levels positively correlated with the latency times.

Activation of the apoptosis pathway promotes dopaminergic cell death in the SNpc34. The BCL2 family proteins, BAX and BCL-2, are key regulators of mitochondria-mediated intrinsic apoptosis, and their levels control the release of the proapoptotic protein cytochrome c into the cytosol, which then activates caspase-9 and -3 in an orderly fashion to execute apoptosis35. Consistent with the recent results13, the present data also showed that MPTP/P treatment upregulated BAX and downregulated BCL-2, thus resulting in an abundance of active caspase-3. In contrast, TE reversed the MPTP-induced proapoptotic-prone environment to an anti-apoptotic milieu by reducing BAX and increasing BCL-2. These results suggested that the TE-induced anti-apoptotic effects were associated with an attenuation of dopaminergic cell loss in the SNpc.

## CONCLUSIONS

The present results demonstrated that TE restored motor function and preventedthe dopaminergic neuronal loss in the SNpc caused by pharmacologicallyinduced PD. In addition, the TE-induced autophagy was not a result ofdisrupted fusion processes between autophagosomes and lysosomes. Instead, it was a result of enhanced autophagy flux, which was evidenced by the decreased levels of p62 and increased levels of LAMP2 expression. Taken together, the present results suggested that TE-related increases in autophagyare important mechanisms in exercise-induced neuroprotection against PD. This finding provides some insights for the development of potential therapeutic strategies that target autophagy to limit the pathogenesis and progression of PD.
